# Arousal, subjective significance and the origin of valence aligned words in the processing of an emotional categorisation task

**DOI:** 10.1371/journal.pone.0265537

**Published:** 2022-03-31

**Authors:** Kamil K. Imbir, Joanna Duda-Goławska, Adam Sobieszek, Adrianna Wielgopolan, Maciej Pastwa, Jarosław Żygierewicz

**Affiliations:** 1 Faculty of Psychology, University of Warsaw, Warsaw, Poland; 2 Biomedical Physics Division, Institute of Experimental Physics, Faculty of Physics, University of Warsaw, Warsaw, Poland; Universidad Complutense Madrid, SPAIN

## Abstract

An emotional categorisation task allows us to study how emotionality is understood and how emotional factors influence decisions. As emotionality is not only the valence but is also composed of activation (arousal and subjective significance) and the type of process needed to produce emotion (origin), we wanted to test the influence of these emotional factors on with a group of stimuli not differing in valence. We predicted that increasing activation levels should lead to increased classification of stimuli as emotional, with a focus on the late processing stages, when explicit word processing occurs, which on the electrophysiological level corresponds to P300, N450 and LPC components. The behavioural results showed that the emotionality of words increased with increasing levels of arousal and subjective significance. Automatically originated words were assessed as more emotional than reflective ones. The amplitude of the N450 component revealed dissociation for subjective significance and origin effects, showing that these two dimensions ascribe distinct properties of emotionality. Finally, the LPC component was susceptible to all affective dimensions used in manipulation. Our study showed that arousal, subjective significance and origin are dimensions of affect that shape the processing of words’ emotionality, when the values of valence were aligned among the stimuli.

## 1. Introduction

### 1.1. Emotional factors

Assessing the emotionality of a stimulus is one of the most basic cognitive processes for the interpretation of the external world. Many factors contribute to the emotional value of stimuli, three of which were manipulated in the presented study. Arousal is the factor that indicates the intensity of the emotional reaction to a stimulus [1]. A highly arousing charge means that a stimulus evokes a strong and intense emotional reaction. It is important to note that the arousing value may be evoked by pleasurable stimuli (such as sexual ones or those related to reward), but also threatening ones or those related to action or movement, which are rather moderate in the case of pleasure. A high arousal load usually disrupts complex cognitive processes, thus lengthening the reaction times [2, 3]. However, arousal may also interact with emotional valence of stimuli the dimension indicating the stimuli being positive or negative. High arousal may speed up processing of strongly negative words [4, 5].

Another factor is subjective significance [6, 7], which means the importance of a certain thing or idea to a person. There are individual differences between what is important to a person, but within a certain society, high significance tends to be related to particular ideas. Highly significant stimuli could be ones related to personal life (such as family or childhood), but also important concepts, such as religion or ethics [8–10]. The hypothesis of the duality of emotional functioning suggests that subjective significance influences effortful (reflective) processing just as arousal influences effortless (automatic) processing. For example, in a cognitive control domain, an increased subjective significance level was found to shorten reaction times [7, 11, 12].

The last interesting emotional dimension is the origin, which is a dualistic concept related to the automatic vs reflective mechanisms for emotional reaction formation [9, 13, 14]. The automatic emotions are derived from biological processes, such as pain or hunger; they cause reactions that are fast and innate, effortless even. An example of such emotion might be fear as an answer to some frightening external stimuli—we feel terrified automatically, as this is an evolutionary mechanism ensuring fast reaction. The reflective emotions are related to more complex social concepts, such as achieving one’s goals or being approved by society; reflective emotions are tied with the already gained knowledge, understood standards and rules [9]. They are slower, more effortful (requiring some cognitive processing and intentional thinking about the situation), and easier to verbalize [15]; an example would be the feeling of shame after doing something embarrassing. Compared to the aforementioned fear, the emotion of shame appears slower, and it requires cognitive processing—understanding the social rules and the situation of not obeying them. Earlier studies have suggested that these origins have shaped how we process information [16–19]. In the current experiment we decided to align to these emotional factors in terms of valence, which is one of the most basic emotional factors and is most likely to interact with other emotional factors [20].

### 1.2. Emotional word processing stages: EEG

Emotional word processing from the event-related potential (ERP) perspective can be studied from two approaches—that is, with a focus on involuntary or voluntary processing [21, 22]. The first employs tasks such as the Emotional Stroop Task [12, 23, 24], in which we observe modulation occurring mostly in early components associated with automatic processing and conflict resolution [25–27]. The second type employs tasks that require explicit, deeper processing of words, in which we observe modulation on later components associated with semantic analysis of the words’ meaning [28] and attention to task demands [29, 30]. The Emotional Decision Task—which we used in our study—is a paradigmatic task of the second kind. In this task, participants are presented a single word on a screen. With each stimulus they are asked to make a simple decision, indicating (usually with the use of keyboard key) whether it is an emotional or non-emotional word. Emotional Decision Task requires a deeper, semantic processing of words from participants and an explicit assessment of their emotionality, which might be very much affected by characteristics of the presented words. Thus, we consider in more detail the later components of interest in such a case: P300, N450 and LPC.

The first of these which we studied was P300, which is localised in the parieto-central regions on a scalp and peaks in the time window between 250 and 350ms [31]. P300 is usually present in paradigms in which the differentiation between target and standard stimuli plays a role, such as in an oddball task paradigm [32]; similarly, significant differences were observed between emotional and non-emotional stimuli, with the former causing more positive amplitudes than the latter. P300 amplitudes are also sensitive to the importance of the stimuli (namely, the more meaningful, important stimuli elicit more positive amplitudes [24, 33]). It has been suggested [34] that the P300 amplitude could be changed not only by the meaningfulness of the stimuli (and how relevant it is to the current, updated context of the task [30]) but also by the assessment of the further consequences and implications of the stimuli.

The N450 component is a negative peak, present about 350–500 ms after stimulus onset. It is usually associated with the frontocentral regions on a scalp, but can also occur as a rather broadly distributed negativity [35]; the changes in the amplitude of N450 are associated with activity of the anterior cingulate cortex [36, 37]. N450 reflects dealing with conflict, meaning both the processes of detecting [38] and monitoring conflict, as well as conflict resolution, cognitive control (and its necessary adjustments caused by the conflict) and engaging cognitive inhibition in making a decision between two competing reactions [32, 36, 39]. The amplitude of N450 was also verified to be susceptible to the subjective significance of the stimuli; namely, words of low subjective significance caused more negative potentials than those of medium or high significance [11, 12].

The last component is the late positive complex (LPC). Its amplitude peaks between 500 and 800 ms, and it is located in the posterior area [21]. The LPC is a manifestation of later stages of semantic processing [40, 41] associated with conscious recognition of and attention to stimulus [30], so it has been shown to become more emotionally modulated as the level of attention to the word’s emotionality increases [22, 42, 43]. This could explain the inconsistent results regarding LPC effects seen in early studies of emotional word processing (e.g. [25–27, 44–46]). However, as emotionality may be measured on many theoretical dimensions, emotionality itself may be said to possess an internal structure. Thus the next step for research is to move beyond the negative-positive distinction and differentiate this structure at the level of ERPs. Hinojosa, Moreno and Ferré [47] acknowledged origin as one of the factors pertinent for future neurolinguistic study, while our previous studies showed that arousal and subjective significance, as the two aspects of emotional activation, evoke distinct patterns of ERP modulation [12, 48].

### 1.3. Aim and hypothesis

The current experiment sought to determine the role of activation dimensions (arousal, subjective significance) and the origin of affective state in explicit processing of emotionality. Because emotions are understood not only in the context of valence but also activation (defined often as arousal: [1]), it is important to know how arousal shapes the neural correlates of emotional categorisations in neutrally valenced stimuli. In the current experiment, we approached the issue from the dual-mind perspective [6, 49], distinguishing stimuli of different origins (automatic vs reflective) and comparing the effects of arousal to the effects of subjective significance. We expected, on a behavioural level, that increasing activation level (arousal and subjective significance) would lead to the increasing classification of stimuli as emotional. On the electrophysiological level, due to the exploratory nature of this study (three dimensions crossed orthogonally for the first time), we formulated only a few hypotheses. Considering the N450 component, we expected increasing levels of arousal to make amplitudes more negative, while increasing levels of subjective significance would make amplitudes less negative [12, 50]. In the LPC component, which is associated with meaning processing [21], we expected to find main effects for all emotional variables. Such patterns should indicate the explicit discrimination of all variables.

## 2. Materials and methods

### 2.1 Participants

The experimental group consisted of 36 participants (18 women and 18 men), aged from 19 to 32 years old (*M* = 24.03, *SD* = 3.26). Participants were students in different faculties at Warsaw universities. The participants were all native Polish speakers, right-handed, with intact vision or corrected to normal by glasses. Additionally, none of the participants had a clinical issue or took medication that could disrupt the EEG recordings. All participants received remuneration for taking part in the experiment. After preprocessing the data, we removed two participants from the experimental group, as their EEG recordings did not meet the quality criteria we adopted (the criteria are further described in section 2.5.2). The final sample consisted of 34 participants, 16 women and 18 men, aged from 19 to 32 years old (*M* = 24.09; *SD* = 3.34).

We did not collect any personal data from the participants that would allow their identification. The participants provided informed consent to participate in the experiment, which was documented in a research diary. The bioethical committee at the Faculty of Psychology, University of Warsaw, approved the design, experimental conditions and procedure. All of the procedures involving human participants were completed in accordance with the institutional and national research committee’s ethical standards and with the 1964 Helsinki declaration and its later amendments or comparable ethical standards.

### 2.2 Design

The independent variables were divided and crossed in the following manner: 3 levels of arousal (low vs medium vs high) × 3 levels of subjective significance (low vs medium vs high) × 3 levels of origin (automatic vs no particular origin vs reflective), thus constituting 27 emotional conditions. Valence, frequency of appearance in language and length of words were aligned and controlled. The dependent variables were behavioural and EEG measures related to the interference of cognitive control.

### 2.3 Linguistic materials

Words used in the present study were acquired from the Affective Norms for Polish Words Reloaded database [51]. This dataset provides scores for eight different affective measures for 4,900 Polish words. Of these, origin, subjective significance, arousal and valence were the dimensions of interest. In the study establishing said database, each word was rated on each dimension by 50 participants, half of whom were men, using a 9 point SAM scale (the mean ratings reported later are for values between 1 and 9). The ratings were then converted into means for every scale.

We selected words in 27 groups (3 × 3 × 3 design, 15 words in each group), differing in levels of arousal (low, medium, high), origin (automatic, null, reflective) and subjective significance (low, medium, high), but not in any of the controlled factors. We controlled for valence, word length (number of letters) and frequency of use [52]. All of the words chosen were nouns. Adjectives were excluded because Polish adjectives vary in form depending on the grammatical gender of the noun to which they refer; the affective norms database we used also only contained adjectives in their masculine form, so possible issues stemming from this fact could not be controlled for.

In our final list of 405 words, mean ratings when dividing by the three levels of arousal were *M* = 3.38, *SD* = 0.32 for words with low arousal; *M* = 4.09, *SD* = 0.27 for moderately arousing words; and *M* = 4.87, *SD* = 0.36 for highly arousing words. Mean ratings for groups divided by origin were *M* = 4.65, *SD* = 0.38 for automatic; *M* = 5.46, *SD* = 0.28 for null; and *M* = 6.41, *SD* = 0.40 for reflective stimuli. Last, for the dimension of subjective significance, the ratings were *M* = 3.12, *SD* = 0.33 for low; M = 3.81, SD = 0.27 for medium; and *M* = 4.80, *SD* = 0.42 for highly significant words. Descriptive statistics for all words and the 27 experimental groups are reported in S1 Appendix.

We performed a 3 (arousal) × 3 (origin) × 3 (subjective significance) ANOVA analysis to verify our selection for each of the six variables (3 manipulated and 3 controlled), treated as dependent variables. A valid selection would yield only three significant effects—namely, an effect of arousal on arousal ratings, of origin on origin ratings and of significance on significance ratings. The data for each word’s frequency of use were transformed into natural logarithms to more closely approximate a normal distribution.

Arousal ratings differed significantly between groups divided by arousal: *F*(2, 378) = 725.74, *p* < 0.001, *η*^*2*^ = 0.79; but this was not the case for subjective significance *F*(2, 378) = 0.24, *p* = 0.77, *η*^*2*^ = 0.001; or for origin *F*(2, 378) = 1.08, *p* = 0.34, *η2* = 0.006. As for controlled dimensions, groups of different arousal did not differ in terms of valence ratings: *F*(2, 378) = 2.66, *p* = 0.071, *η*^*2*^ = 0.014; or in terms of word length: *F*(2, 378) = 0.65, *p* = 0.56, *η*^*2*^ = 0.003; or frequency of use: *F*(2, 378) = 1.56, *p* = 0.21, *η*^*2*^ = 0.008.

For groups divided by origin, there were significant differences in origin ratings: *F*(2, 378) = 826.05, *p* < 0.001, *η*^*2*^ = 0.81; but not in arousal ratings: *F*(2, 378) = 1.19, *p* = 0.31, *η*^*2*^ = 0.006; or in ratings of subjective significance: *F*(2, 378) = 0.13, *p* = 0.88, *η*^*2*^ = 0.001. There were also no differences in terms of valence ratings: *F*(2, 378) = 1.38, *p* = 0.25, *η2* = 0.007; the number of letters: *F*(2, 378) = 0.40, *p* = 0.67, *η*^*2*^ = 0.002; or frequency: *F*(2, 378) = 0.37, *p* = 0.69, *η*^*2*^ = 0.002.

Finally, the analyses showed differences in subjective significance ratings for groups divided by subjective significance: *F*(2, 378) = 807.10, *p* < 0.001, *η*^*2*^ = 0.81. The groups did not, however, differ in arousal ratings: *F*(2, 378) = 0.72, *p* = 0.49, *η*^*2*^ = 0.004; or in ratings of origin: *F*(2, 378) = 0.39, *p* = 0.68, *η*^*2*^ = 0.002. There were also no differences in valence ratings: *F*(2, 378) = 2.66, *p* = 0.071, *η*^*2*^ = 0.014; word length: *F*(2, 378) = 1.86, *p* = 0.16, *η*^*2*^ = 0.01; or frequency of use: *F*(2, 378) = 1.31, *p* = 0.27, *η*^*2*^ = 0.007. Moreover, we found no significant interaction effects, as none of the three possible two-way interactions between manipulated factors (arousal and subjective significance, origin and subjective significance and origin and arousal) were statistically significant, either for manipulated or controlled dimensions. Neither was there a three-way interaction between arousal, origin and subjective significance for any of the manipulated or controlled scales. All 405 words used in the experiment—as well as their affective measures, length and frequency of use—can be found in S1 Appendix.

### 2.4 Procedure

All of the participants were seated in a comfortable chair, about 1 m from the computer screen. The stimuli were presented in Helvetica font, taking 10% of the screen height (17.3 inch LCD). The instructions asked participants to react to the stimuli as quickly and accurately as possible. Participants were asked to fulfil the Emotional Decision Task and decide whether the word presented on a screen was emotional or non-emotional. They responded by pressing one of the specifically tagged keys on the keyboard, allowing us to gather data about both the content and the exact timing of the response.

**Fig 1:**
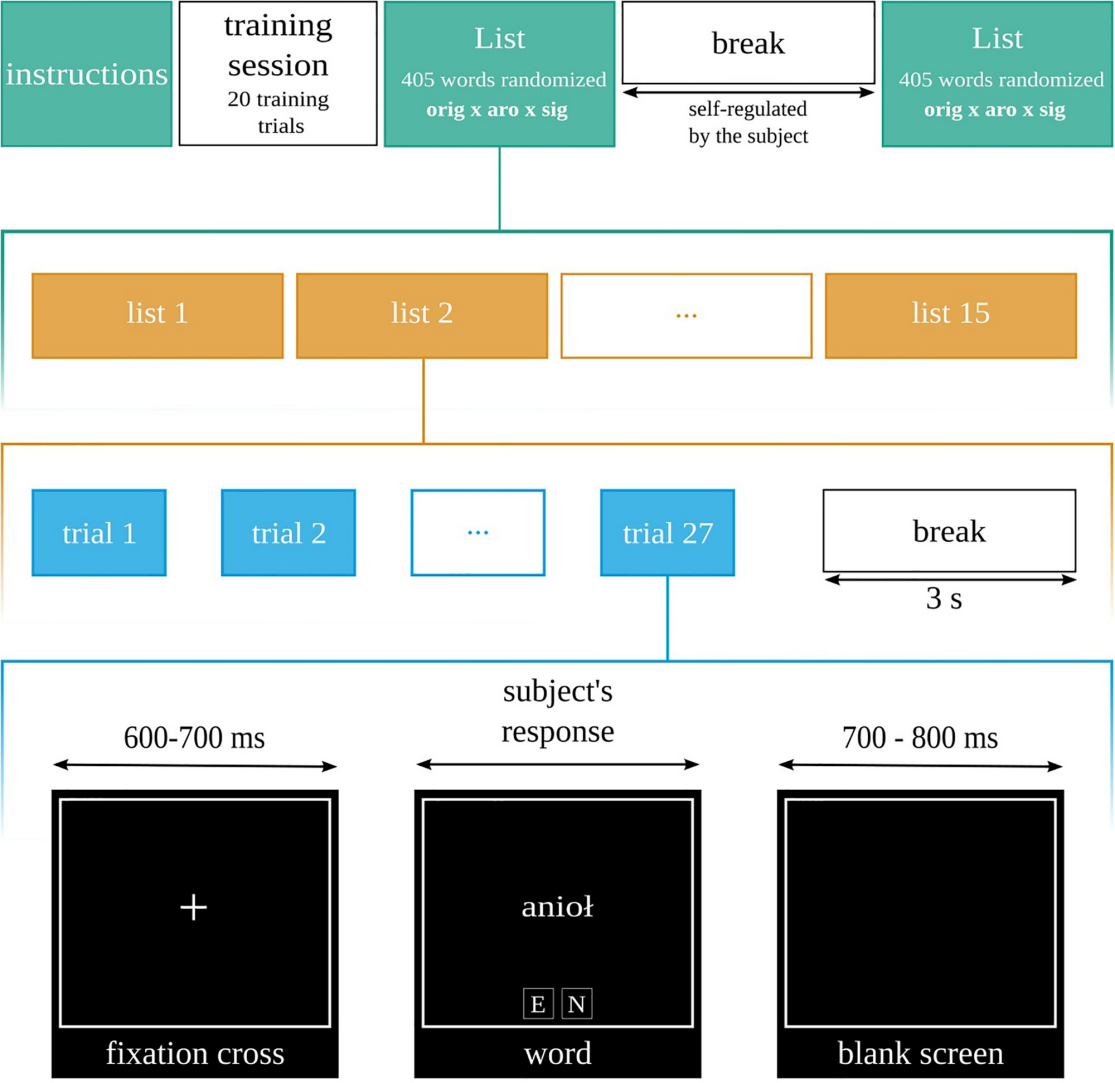
Diagram of the experimental protocol. The task was to assess if the word was emotional or neutral.

The experimental block consisted of 405 word stimuli randomly displayed and was repeated twice in the whole procedure. For each trial, the sequence was the same: first, participants saw the fixation cross (presented for a randomly assigned time interval between 600 and 700 ms); after that, they were presented with a word (presentation was terminated when participant provided an answer; however, it could not be shorter than 300 ms); after the response, the blank screen was shown for 600–700ms (randomised in the same method as for the time interval for fixation cross). After every 27 trials, participants could rest and blink for three seconds. The whole procedure is presented in Fig 1.

### 2.5 EEG recording

#### 2.5.1 Apparatus

The stimuli were displayed on a standard PC monitor. The stimuli were synchronised to EEG data using a custom-built circuit that recorded the brightness of a small square on display, hidden from the subject’s sight. Its illumination varied synchronously with the content of the display. We registered EEG signal from 19 electrode sites: Fz, Cz, Pz, Fp1/2, F7/8, F3/4, T7/8, C3/4, P7/P8, P3/4, O1/2 referenced to linked earlobes and grounded at the AFz position. All impedances were kept at a comparable value under 5 kOhm. The signal was obtained using a Porti7 (TMSI) amplifier, sampled at 1024 Hz.

#### 2.5.2 Offline EEG signal processing

We carried out the offline signal processing using MATLAB^®^ with the EEGLAB toolbox [53] and custom-made codes. Offline, the signal was zero-phase filtered with the high-pass cutoff 0.1Hz, the low-pass cutoff 30 Hz, the notch for the 49.5–50.5 Hz band, all implemented as second-order Butterworth filters with 12 dB/octave roll-off. We extracted intervals ranging from −200 to 800 ms, with 0 being the onset of the stimulus. Next, the signals were baseline corrected to the −200–0 ms interval.

We excluded trials shorter than t (Q1 − W) or longer than (Q3 + W), where Q1 is the 25th percentile, Q3 is the 75th percentile and W = 1.5*(Q3 − Q1), computed individually for each subject on the logarithm transformed latencies. The response time for the analysed data across all the subjects was within the range of 70 to 8398 ms.

In the EEG data, channels with kurtosis exceeding 5 were deleted and then interpolated with the *interpol*() function. Trials with an excessive linear trend (threshold slope value exceeding 30) or excessive amplitude (out of range −65uV 65uV) were marked as corrupted by artefacts and removed from the analysis. Subjects that had more than 50% of trials identified as corrupted were eliminated from further research. In the analysed data, the mean number of trials per condition was *M* = 26.9, *SEM* = 0.1.

### 2.6 Statistical procedures

We investigated the logarithm of reaction time, emotionality ratings and the EEG component amplitude (the average amplitude within the characteristic time-window and region of interest) using ANOVA with repeated measures in a hierarchical procedure. The significant main effects were further analysed with post-hoc paired t-tests with Holm’s correction for multiple comparisons [54]. The significant two-way interactions were investigated further, using a series of one-way ANOVA with repeated measures within individual levels of the interacting factors, followed by post-hoc paired t-tests with Holm’s correction. We corrected the significance of the effects repeatedly appearing in the given series of ANOVAs by Bonferroni correction. In the case of significant three-way interactions, we continued the tests using a series of two-way ANOVAs with the levels of a chosen variable set iteratively to subsequent levels. The chosen variables were permuted. Obtained significant two-way interactions were further investigated using post-hoc t-tests with Holm’s correction. If different paths in the hierarchical analysis could reach an effect, we report the most conservative result. We applied Greenhouse–Geisser correction where the sphericity assumption was violated according to Mauchly’s test. We used procedures implemented in the r statistical package [55].

## 3. Results

### 3.1. Reaction time

We investigated the reaction time (RT) dependence on the origin, arousal and subjective significance using three-way ANOVA with repeated measures. We used natural logarithm transformations to make the distribution of response latencies closer to normal. In this section, we report the values of *M* and *SEM* in milliseconds.

**Fig 2:**
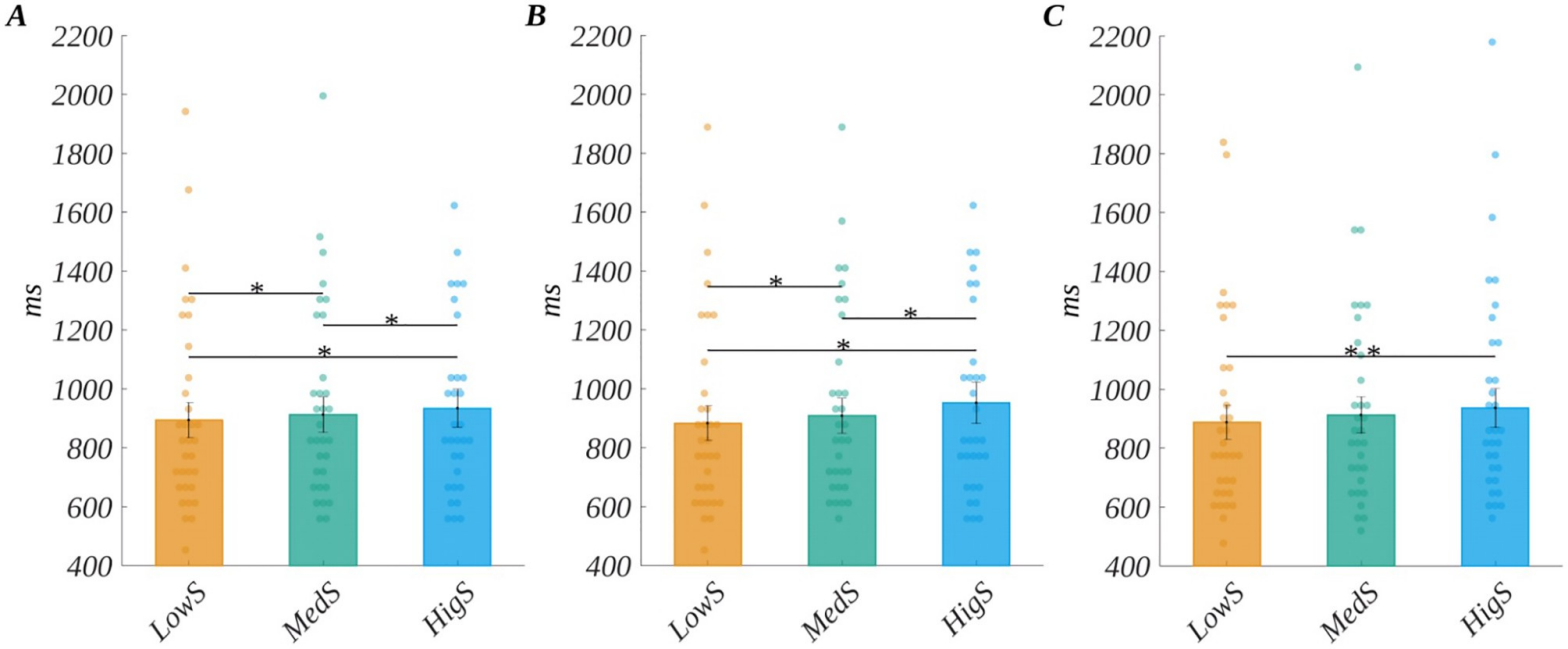
Effects concerning reaction time. (A) The main effect of subjective significance. (B) Interaction between origin and subjective significance for words of no specific origin (Null). (C) Interaction between origin and subjective significance for reflective words. Bars represent the mean value, error bars SEM, individual dots mark the average response time of an individual subject in the given condition, black horizontal lines indicate pairs of conditions with significantly different means (** *p* < .01, * *p* < .05).

The main effect of subjective significance (*F*(1.71, 56.29) = 10.87, *p* < .001, *η*_p_^2^ = 0.25) was found. Mauchly’s test indicated that the assumption of sphericity had been violated for this factor (Xi2 (2) = 0.83, *p* = .048), so degrees of freedom were corrected using Greenhouse-Geisser estimates of sphericity (Epsilon = 0.85). The post-hoc tests showed that the reaction time increased with the level of significance (Fig 2A). Namely, RT for medium significant words (*M* = 912.28, *SEM* = 60.46) was longer than for low significant words (*M* = 893.34, *SEM* = 59.55; *t*(33) = 2.57, *p* = .024, *d* = 0.90); for highly significant words (*M* = 934.07, *SEM* = 65.49), it was longer than for both medium significant (*t*(33) = 2.66, *p* = .024, *d* = 0.93) and low significant (*t*(33) = 3.94, *p* = .001, *d* = 1.37). The other two investigated factors’ main effects were not significant: for origin *F*(2, 66) = 0.62, *p* = .54; *η*_p_^2^ = 0.02; and for arousal *F*(2, 66) = 0.49, *p* = .60; η^2_p = 0.01).

Additionally, we observed the interaction effect between origin and subjective significance (*F*(4, 132) = 3.62, *p* = .008, *η*_p_^2^ = 0.10). For no specific origin, the relation between significance levels follows exactly the one for the main effect of subjective significance, i.e., the reaction time increases with the level of significance, and tests for all pairs of levels are statistically significant. In case of reflective words, we obtained that only the highly significant words correspond to longer reaction times than low significant ones. The reaction times for other pairs of levels do not differ significantly. Finally, for stimuli with automatic origin we did not obtain any significant differences in the reaction times. This effects is illustrated in Fig 2B and 2C, and the details of the statistical tests are presented in S2 Appendix.

### 3.2. Emotional ratings

We analysed the influence of the design factors on the perception of stimuli as emotional by the participants. The frequency at which the stimuli of the given arousal, subjective significance and origin were classified as emotional will hereafter be called emotional rating (ER). We used three-way ANOVA with repeated measures to evaluate the results. We obtained significant main effects for all three factors.

**Fig 3:**
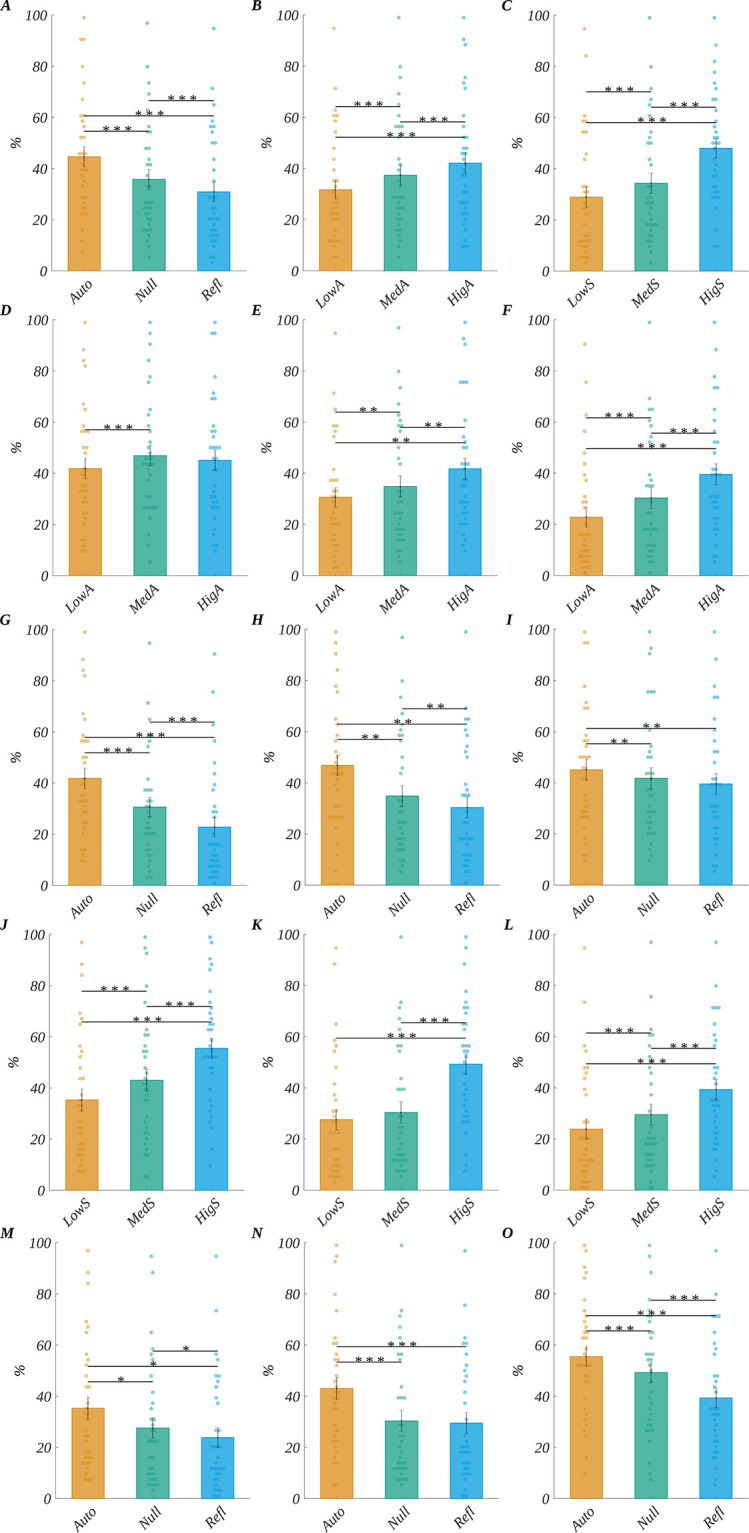
Decision results. The main effects for emotional ratings: (A) origin, (B) arousal, (C) subjective significance. Effects of interactions for emotional ratings: interaction between origin and arousal for words of origin: (D) automatic, (E) Null, (F) Reflective; interaction between origin and arousal for words of arousal level: (G) low, (H) medium, (I) high; interaction between origin and subjective significance for words of origin: (J) automatic, (K) Null, (L) Reflective; and interaction between origin and subjective significance for words of subjective significance level: (M) low, (N) medium, (O) high. Bars represent the mean value, error bars SEM, individual dots mark the average ER of an individual subject in the given condition, black horizontal lines indicate pairs of conditions with significantly different means (* *p*<0.05, ** *p*<0.01, *** *p* < .001).

We obtained the main effect of origin (*F*(1.14, 37.73) = 45.83, *p* < .001, *η*_p_^2^ = 0.58); Mauchly’s test indicated that the assumption of sphericity had been violated for this factor (Xi2 (2) = 0.25, p < .001), so degrees of freedom were corrected using Greenhouse-Geisser estimates of sphericity (Epsilon = 0.57). The emotional ratings decreased with the increase of reflectiveness of the stimuli (Fig 3). The post-hoc tests showed that the ER for words of no specific origin (Null) (*M* = 35.72, *SEM* = 3.81) were significantly smaller than for automatic origin (*M* = 44.60, *SEM* = 3.96; *t*(33) = −8.53, *p* < .001, *d* = −2.97); for reflective words (*M* = 30.89, *SEM* = 3.77) it was significantly smaller than for the null words (*M* = 35.72, *SEM* = 3.81; *t*(33) = −4.21, *p* < .001, *d* = −1.47); also for reflective words (*M* = 30.89, *SEM* = 3.77) ER was smaller than for the automatic stimuli (*M* = 44.60, *SEM* = 3.96; *t*(33) = −6.91, *p* < .001, *d* = −2.41).

Furthermore, the main effect of arousal (*F*(1.20, 39.75) = 24.04, *p* < .001, *η*_p_^2^ = 0.42) was found; Mauchly’s test indicated that the assumption of sphericity had been violated for this factor (Xi2 (2) = 0.34, p < .001), so degrees of freedom were corrected using Greenhouse-Geisser estimates of sphericity (Epsilon = 0.60). The ER increased with the arousal level (Fig 3B). The post-hoc tests indicated that ER for high arousal (*M* = 42.13, *SEM* = 4.04) was significantly bigger than for both medium (*M* = 37.34, *SEM* = 3.87; *t*(33) = 3.91, *p* < .001, *d* = 1.36) and low arousal (*M* = 31.73, *SEM* = 3.64; *t*(33) = 5.15, *p* < .001, *d* = 1.79). It was also bigger for medium than for low arousal (*t*(33) = 5.15, *p* < .001, *d* = 1.79).

Finally, we observed the main effect of subjective significance (*F*(1.14, 37.68) = 53.79, *p* < .001, *η*_p_^2^ = 0.62); Mauchly’s test indicated that the assumption of sphericity had been violated for this factor (Xi2 (2) = 0.25, p < .001), so degrees of freedom were corrected using Greenhouse-Geisser estimates of sphericity (Epsilon = 0.57). The ratings increased with the increase of subjective significance (Fig 3C). The post-hoc tests revealed that ER for highly significant words (*M* = 48.01, *SEM* = 3.77) was bigger than for both medium significant words (*M* = 34.29, *SEM* = 4.00; *t*(33) = 7.54, *p* < .001, *d* = 2.63) and low significant words (*M* = 28.90, *SEM* = 3.96; *t*(33) = 7.52, *p* < .001, *d* = 2.62). Also, ER for medium significant words was bigger than for low significant stimuli (*t*(33) = 5.23, *p* < .001, *d* = 1.82).

We also obtained the interactions between origin and arousal (*F*(2.39, 78.80) = 14.91, *p* < .001, *η*_p_^2^ = 0.31). This effect is illustrated in Fig 3D–3I; the details of the statistical tests are presented in S2 Appendix. The interaction between origin and subjective significance was significant (*F*(4, 132) = 5.78, *p* < .001, *η*_p_^2^ = 0.15). This effect is illustrated in Fig 3J–3O, and the details of statistical tests are presented in S2 Appendix.

### 3.3 EEG

#### 3.3.1. P300

**Fig 4:**
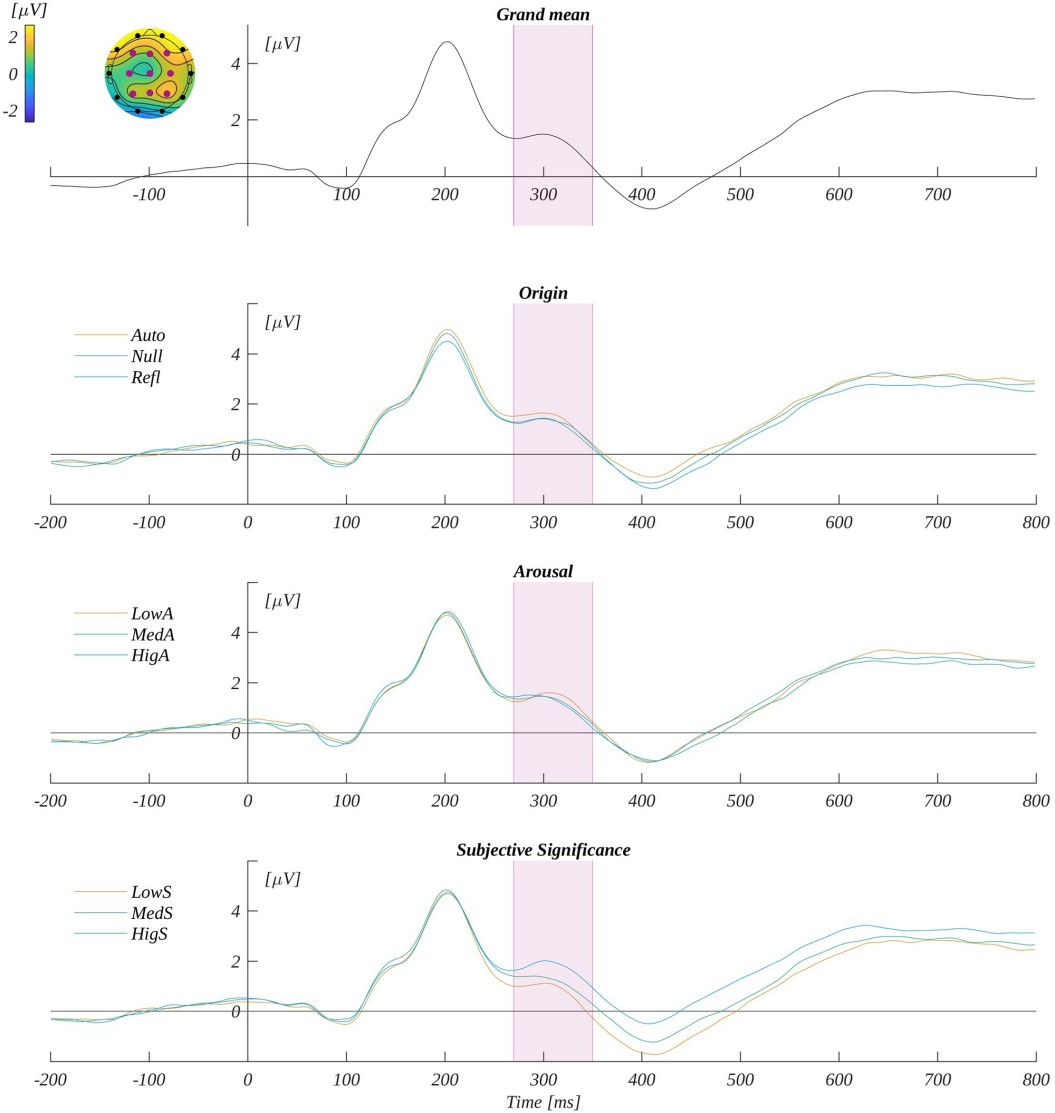
P300 potential. The upper plot: the grand average across electrodes from ROI_P300_. The marked time-interval was selected for the P300 analysis. The insert shows the topography of the mean potential from this period with the electrodes forming the ROI_P300_ marked with pink dots. The three bottom plots are the time courses for ERP averaged across electrodes from ROI_P300_ for each level of origin, arousal and subjective significance.

We analysed the P300 component by averaging the ERP amplitude in the time window 270–350 ms in the characteristic region of interest (ROI_P300_)—that is, F3, Fz, F4, C3, Cz, C4, P3, Pz and P4. The grand average signal from ROI_P300_ and traces for each level of origin, arousal and subjective significance are shown in Fig 4.

**Fig 5:**
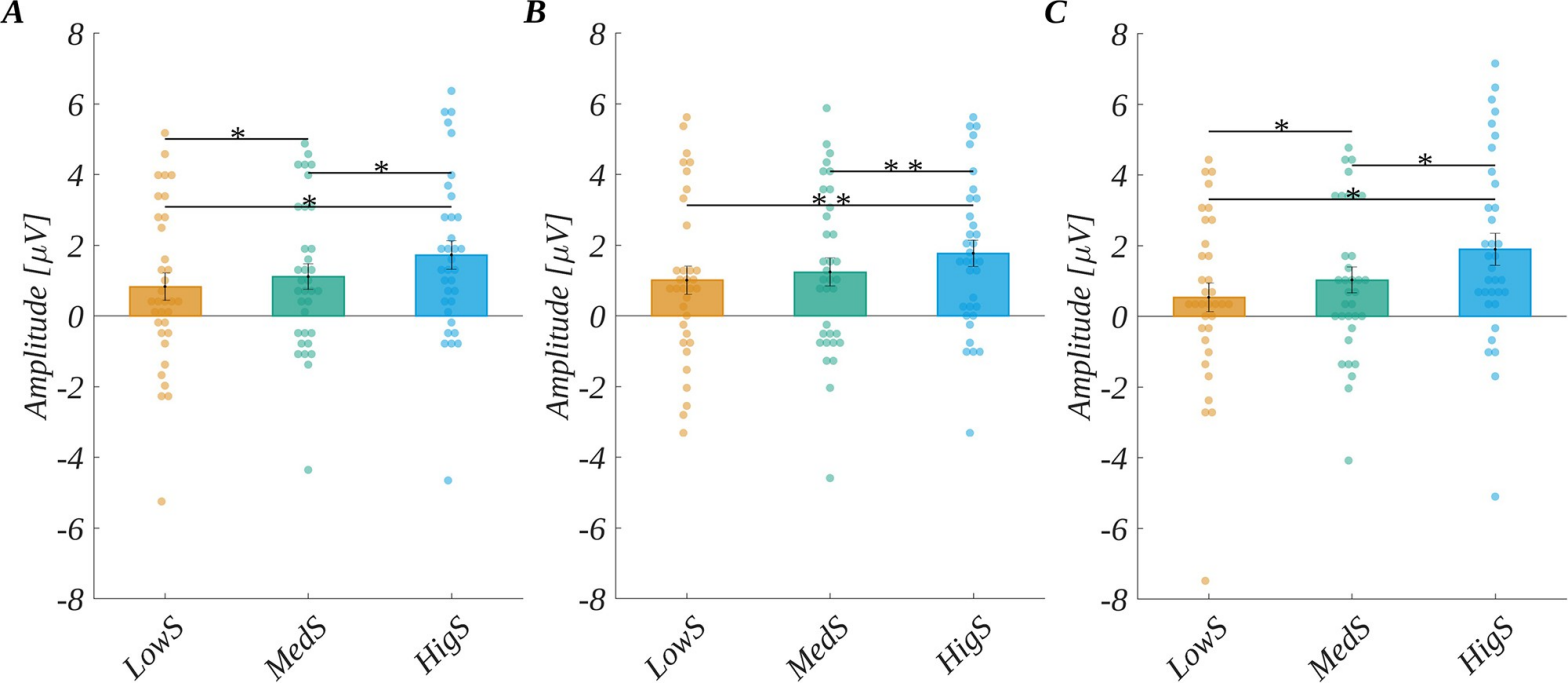
P300 results. The main effect of: subjective significance (A). Interaction between origin and subjective significance: (B) automatic origin and (C) null origin. Bars represent the mean value, error bars SEM, individual dots mark the amplitude for an individual subject in the given condition, black horizontal lines indicate pairs of conditions with significantly different means (* *p* <0.05, ** *p*<0.01).

We observed the main effect of subjective significance (*F*(2, 66) = 29.34, *p* < .001; *η*_p_^2^ = 0.47). The post-hoc tests showed that the amplitude monotonically increased with the level of subjective significance (Fig 5A); namely, it was more positive for medium significant words (*M* = 1.11, *SEM* = 0.36) than for low significant stimuli (*M* = 0.83, *SEM* = 0.39; *t*(33) = 2.28, *p* = .029, *d* = 0.79). Further, it was more positive for highly significant words (*M* = 1.72, *SEM* = 0.40) then for medium significant words (*t*(33) = 5.13, *p* < .001, *d* = 1.79). Finally, it was more positive for highly significant than for low significant words (*t*(33) = 7.78, *p* < .001, *d* = 2.71). The other two design variables’ main effects were not significant (for origin *F*(2, 66) = 2.02, *p* = .14; *η*_p_^2^ = 0.06; or for arousal *F*(2, 66) = 1.08, *p* = .35; *η*_p_^2^ = 0.03). We also obtained the interaction between origin and subjective significance *F*(4, 132) = 2.68, *p* = .035, *η*_p_^2^ = 0.08). For words of automatic origin, the amplitude corresponding to the high subjective significant level was more positive than for both low and medium significant levels. Further, for words of no specific origin the amplitude increased monotonically with the level of subjective significance, and all pairs of comparisons between the levels of subjective significance were statistically significant. These effects are illustrated in Fig 5B and 5C, and the details of the corresponding statistical tests are presented in S2 Appendix.

#### 3.3.2. N450

**Fig 6:**
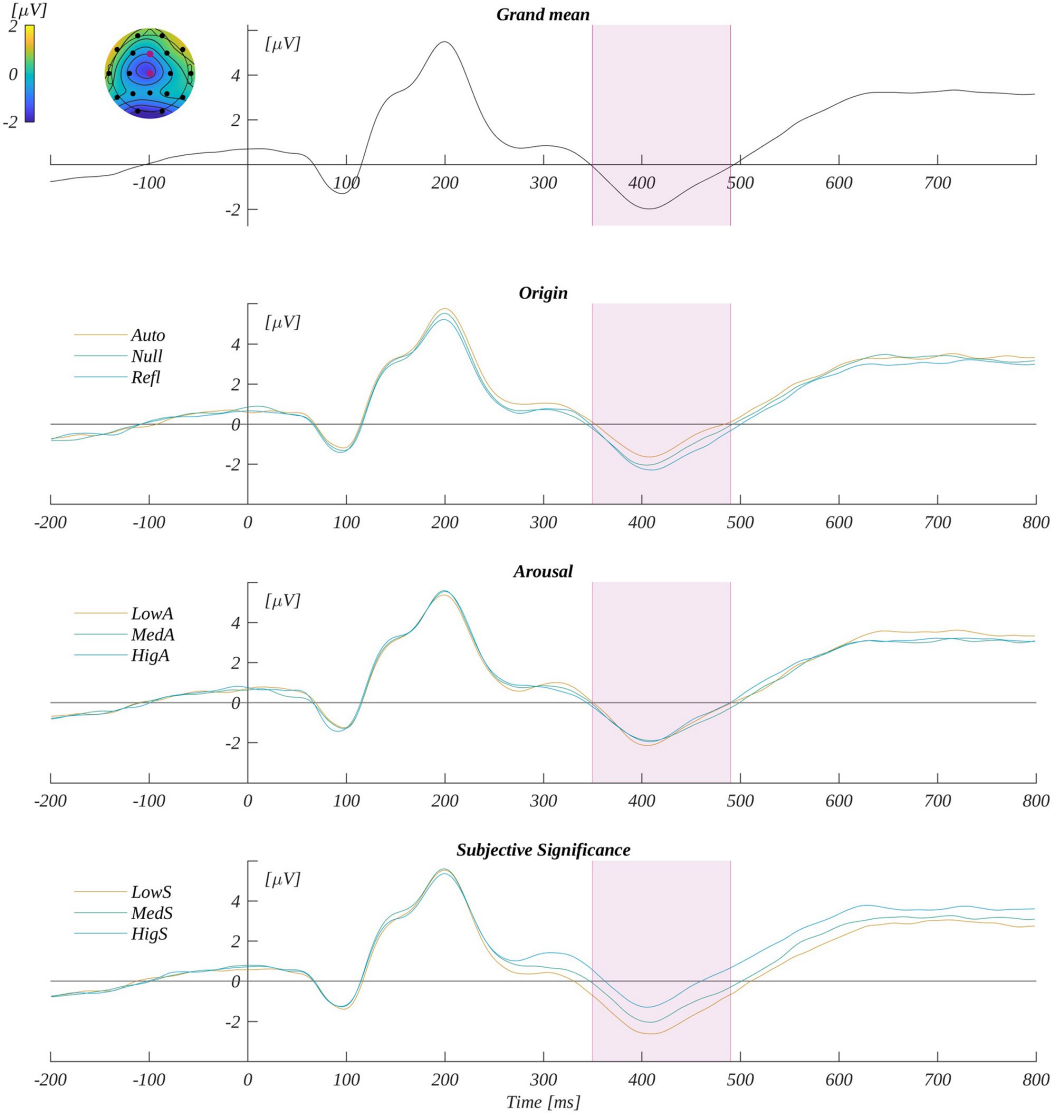
N450 potential. The upper plot: the grand average across electrodes from ROI_N450_. The marked time-interval was selected for the N450 analysis. The insert shows the topography of the mean potential from this period with the electrodes forming the ROI_N450_ marked with pink dots. The three bottom plots are the time courses for ERP averaged across electrodes from ROI_N450_ for each level of origin, arousal and subjective significance.

We analysed the N450 component by averaging the ERP amplitude in the time window 350–490 ms in its characteristic region of interest—that is, Cz and Pz (ROI_N450_). The grand mean from ROI_N450_ and the mean for each level of origin, arousal and subjective significance are shown in Fig 6.

**Fig 7:**
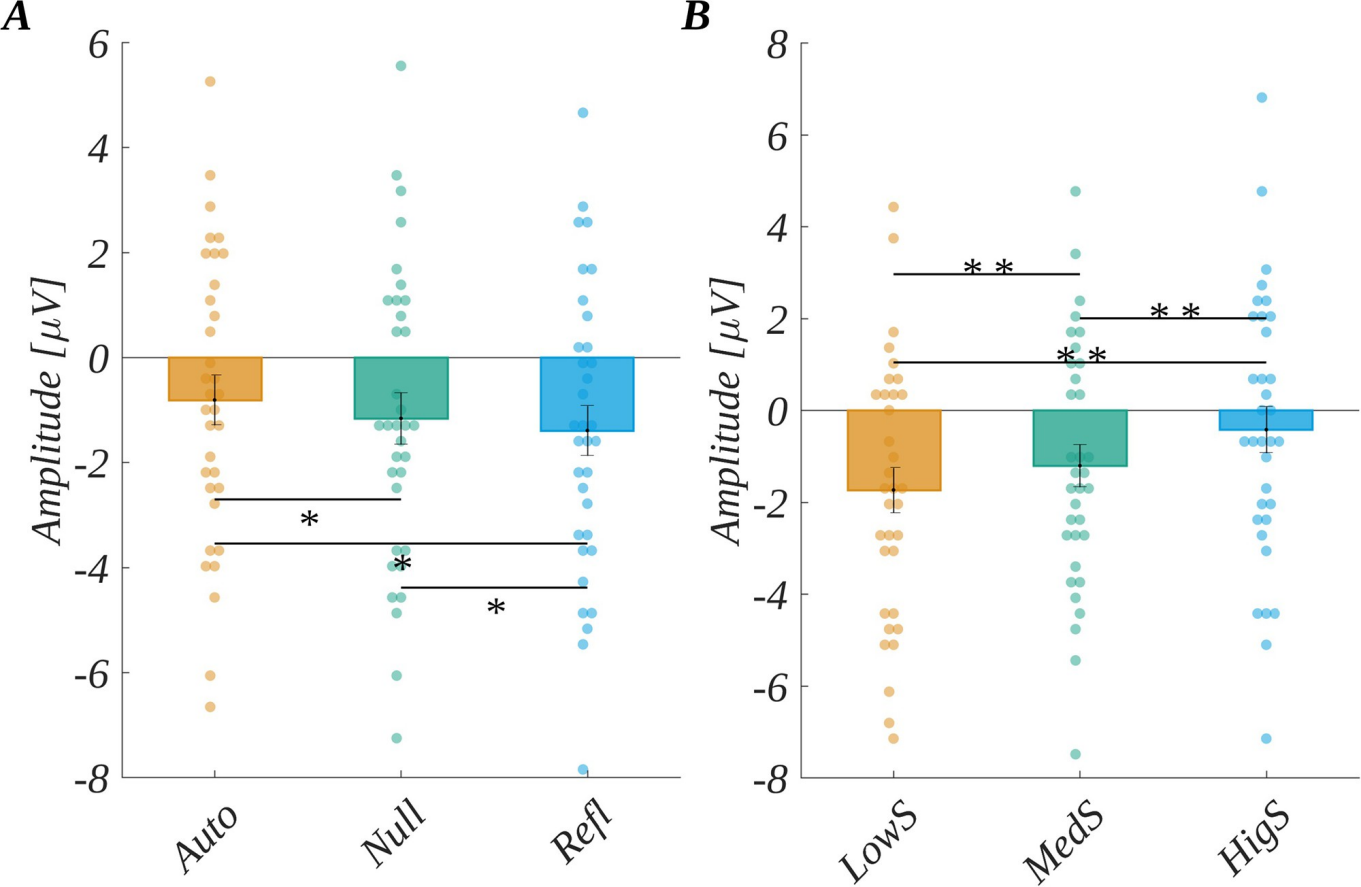
N450 results. The main effect of: (A) origin and (B) subjective significance; Bars represent the mean value, error bars SEM, individual dots mark the amplitude for an individual subject in the given condition, black horizontal lines indicate pairs of conditions with significantly different means (* *p*<0.05, ** *p*<0.01).

In the three-way ANOVA with repeated measures, we obtained the main effect of origin (*F*(2, 66) = 13.78, *p* < .001; *η*_p_^2^ = 0.29; see Fig 7A). The post-hoc tests revealed that the amplitude was progressively more negative as the level of origin changed from automatic through null to reflective. Specifically, the amplitude for automatic words (*M* = −0.81, *SEM* = 0.47) was significantly less negative than for both null words (*M* = −1.16, *SEM* = 0.49); *t*(33) = 3.49, *p* = .003, *d* = 1.22) and reflective words (*M* = −1.39, *SEM* = 0.47); *t*(33) = 4.75, *p* < .001, *d* = 1.65). Also, the amplitude for reflective words was significantly more negative than for null words (*t*(33) = −2.09, *p* = .044, *d* = −0.73).

Moreover, we found the main effect of subjective significance (*F*(2, 66) = 28.52, *p* < .001; *η*_p_^2^ = 0.46; see Fig 7B). Here, the amplitude became less negative with increasing significance of the stimuli. The post-hoc tests showed that the amplitude for highly significant words (*M* = −0.42, *SEM* = 0.50) was less negative than for both the low significant words (*M* = −1.74, *SEM* = 0.49; *t*(33) = −6.89, *p* < .001, *d* = −2.40) and the medium significant words (*M* = −1.21, *SEM* = 0.46; *t*(33) = −4.97, *p* < .001, *d* = −1.73). Finally, the amplitude for medium significant words was significantly less negative than for low significant words (*t*(33) = 3.02, *p* = .005, *d* = 1.05).

The main effect of arousal (*F*(2, 66) = 0.47, *p* = .62; *η*_p_^2^ = 0.01) was not found to be statistically significant. We also found a three-way interaction between origin, arousal and subjective significance (*F*(8, 264) = 2.23, *p* = .026, *η*_p_^2^ = 0.06). An illustration of this effect and the details of the statistical analysis are reported in S2 Appendix. Fig 7 presents the pattern of the results for N450 component.

#### 3.3.3. LPC

**Fig 8:**
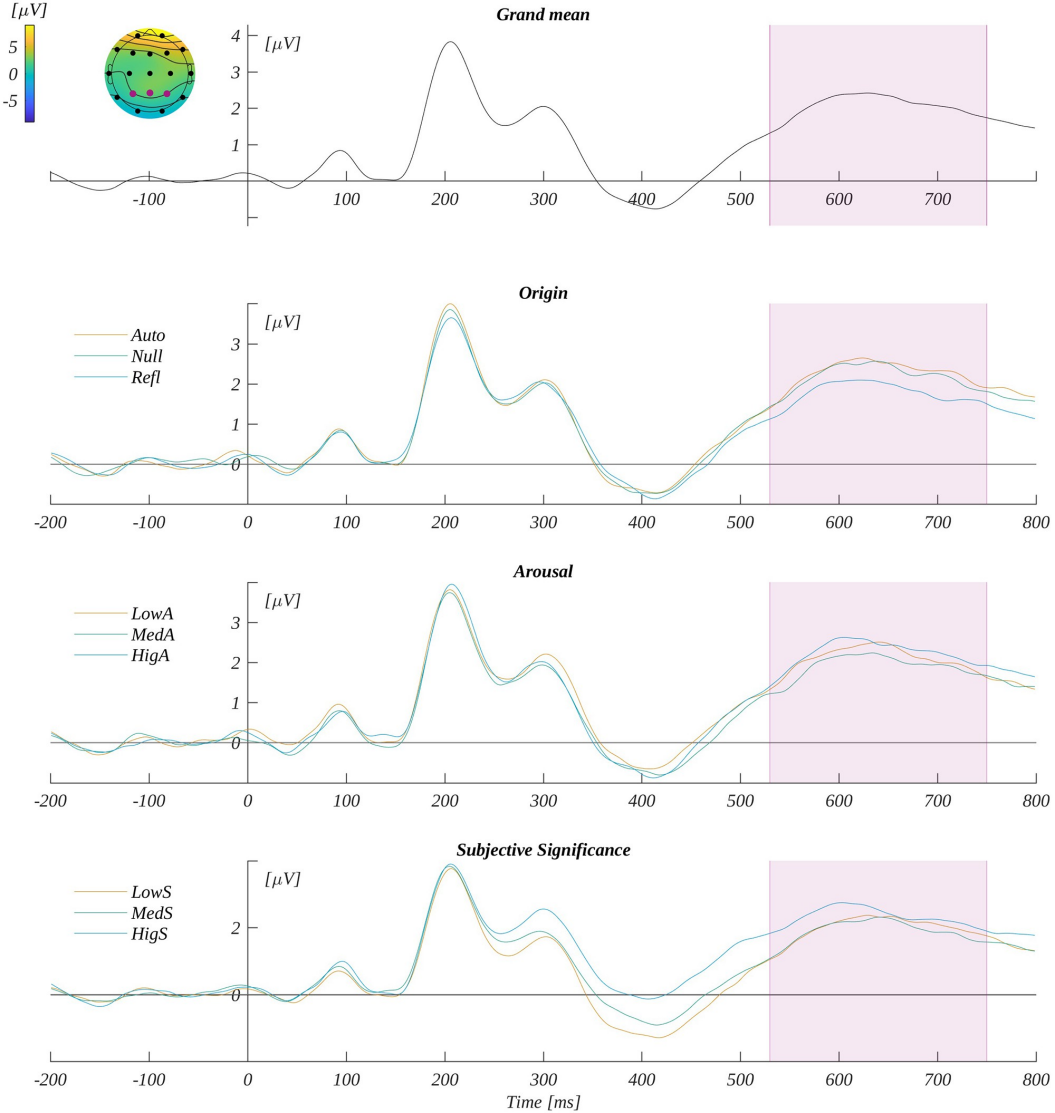
LPC potential. The upper plot: the grand average across electrodes from ROI_LPC_. The marked time-interval was selected for the LPC analysis. The insert shows the topography of the mean potential from this period with the electrodes forming the ROI_LPC_ marked with pink dots. The three bottom plots are the time courses for ERP averaged across electrodes from ROI_LPC_ for each level of origin, arousal and subjective significance.

We analysed the LPC component’s amplitude by averaging ERP in the time window from 530–750 ms and in the region of interest (ROI_LPC_) characteristic for this component—that is, P3, Pz and P4. The grand mean and average signals from ROI_LPC_ for each level of origin, arousal and subjective significance are shown in Fig 8.

In the ANOVA we obtained the main effect of origin (*F*(2, 66) = 8.65, *p* < .001; *η*_p_^2^ = 0.21). The post-hoc tests indicated that the LPC amplitude for reflective words (*M* = 1.81, *SEM* = 0.50) was significantly less positive than for both automatic words (*M* = 2.27, *SEM* = 0.47; *t*(33) = −3.72, *p* = .002, *d* = −1.29) and null words (*M* = 2.21, *SEM* = 0.50; *t*(33) = −3.45, *p* = .003, *d* = −1.20). Further, we found the main effect of arousal (*F*(2, 66) = 4.37, *p* = .016; *η*_p_^2^ = 0.12). Here the post-hoc analysis showed that the amplitude for medium arousal (*M* = 1.93, *SEM* = 0.49) was significantly less positive than for high arousal (*M* = 2.24, *SEM* = 0.49; *t*(33) = −2.90, *p* = .020, *d* = −1.01).

**Fig 9:**
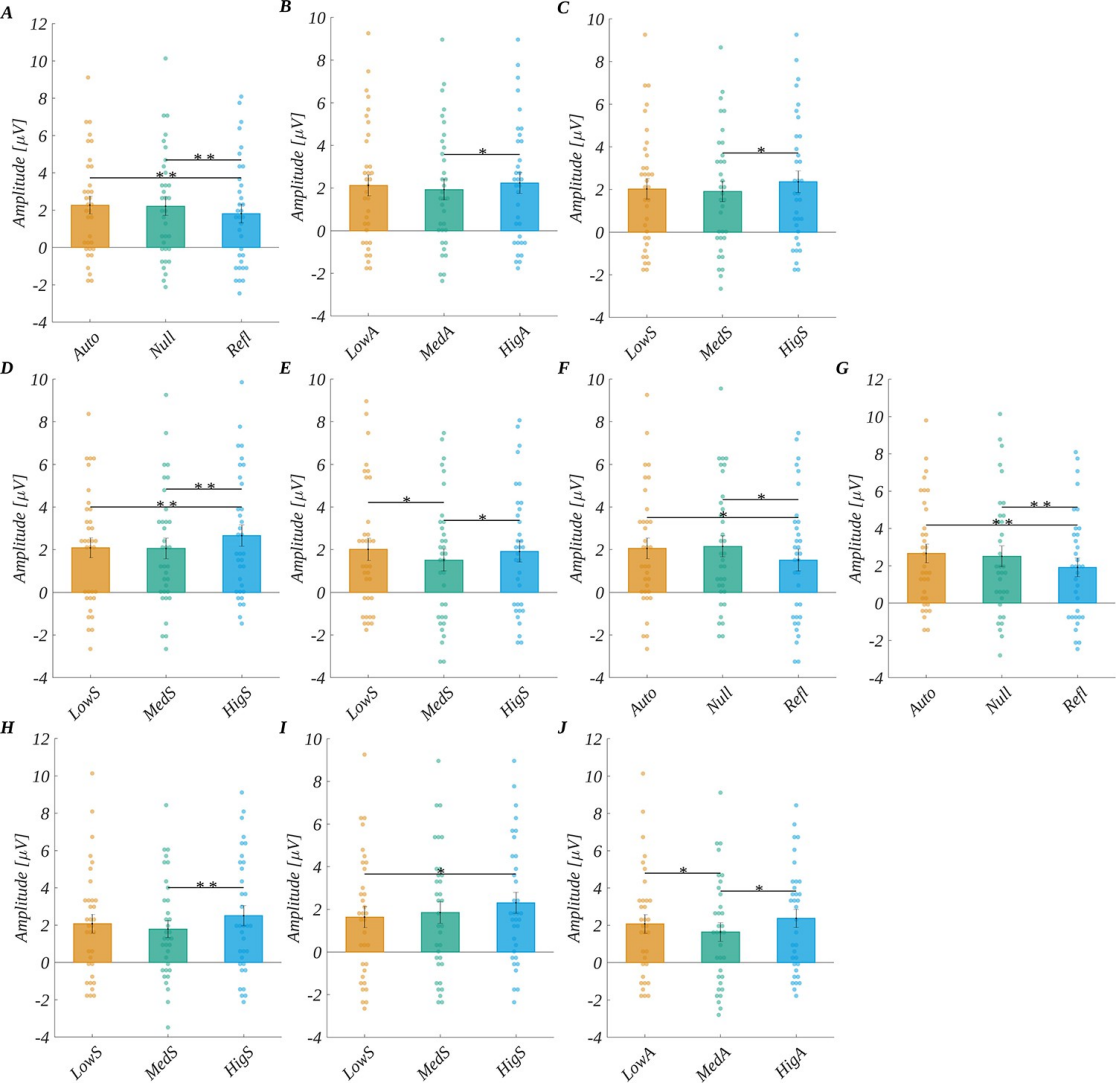
LPC results. The main effect obtained for LPC component (A) origin, (B) arousal, (C) subjective significance. Interaction between origin and subjective significance: amplitude dependence on subjective significance for (D) automatic and (E) reflective origin levels; dependence on origin for (F) medium significant and (G) highly significant stimuli. Interaction between arousal and subjective significance for different levels of subjective significance at (H) low arousal and (I) medium arousal; dependence of the amplitude on arousal for low significant words (J). Bars represent the mean value, error bars SEM, individual dots mark the amplitude for an individual subject in the given condition, black horizontal lines indicate pairs of conditions with significantly different means (* *p*<0.05, ** *p*<0.01).

Finally, the main effect of subjective significance (*F*(2, 66) = 5.95, *p* = .004; *η*_p_^2^ = 0.15) was found to be significant. The post-hoc tests revealed that the amplitude for medium significant words (*M* = 1.91, *SEM* = 0.48) was significantly less positive than for highly significant words (*M* = 2.36, *SEM* = 0.51; *t*(33) = −3.00, *p* = .015, *d* = −1.04). Main effects are presented on Fig 9A–9C.

Additionally, we observed the interaction between origin and subjective significance (*F*(4, 132) = 2.79, *p* = .029, *η*_p_^2^ = 0.08; see Fig 9D–9G). Namely, for the automatic origin we obtained that the amplitude for highly significant words was more positive than for both low and medium significant stimuli. Further for reflective origin, the amplitude for medium significant words was less positive than for both low and highly significant words. On the other hand, for medium significant words the amplitude for reflective stimuli was less positive than for both automatic and null origin stimuli. These relationship holds also for highly significant stimuli. The details of statistical tests’ results are presented in S2 Appendix.

Moreover, we obtained the interaction between arousal and subjective significance (*F*(4, 132) = 2.46, *p* = .048, *η*_p_^2^ = 0.07; see Fig 9H–9J). For low arousing stimuli, we obtained that the amplitude corresponding to the highly significant words was more positive than for medium significant ones. Next, for medium arousing stimuli, we obtained that the amplitude for highly significant words was more positive than for low significant ones. Finally, for low significant words we obtained that the amplitude for medium arousing words was less positive than for both low and highly arousing stimuli. The details of statistical tests’ results are presented in S2 Appendix.

#### 3.3.4. Analyses based on the assessments of emotionality of words

In the next step we wanted to verify the neural correlates of processing depending on the emotionality of words, assessment of which the task was based on. We decided to analyse the data using two approaches: (1) based on emotionality assessments of stimuli taken from a dataset of the normative study (ANPW_R: [51]), and (2) declared emotionality recorded as a decision in the current experiment. For the first approach to these analyses the emotionality of stimuli could be operationalised as the extreme values on the valence dimension assessments (low, negative or high, positive; [51]. Based on this assumption, we classified the words chosen for the experiment as valent (valence rating below 3.5 in the normative study; [51] or valence rating above 6.5), picking the ones between 3.5 and 6.5 mean value of valence as the non-valent words, (cf. S3 Appendix) and conducted ANOVA analyses using this factor. A point worth explaining is the choice of two levels for valence: valenced (either positive or negative) and neutral. This stems from the fact that we made sure to include the same number of positive and negative words in each of the experimental groups. Thus, if any confounding effect of valence was observed in the study, it would have to be evoked by the difference between neutral words and the average effect of positive and negative words. We have to note that if negative and positive valences have opposite effects on ERP amplitudes, then no confounding effect would occur in our study.

The second approach to analyses of emotionality was based on the decisions done by the participants in the present experiments. We include the decisions of participants regarding words (emotional vs non-emotional) as a factor in the ANOVA analyses. We considered the first approach to these analyses to be validation of the experimental design, as the set of words was aligned for valence before the experiment, while the second approach was rather exploratory—we expected distinct effects depending on the type of decision made by the participant. We conducted a total of 18 ANOVA analyses. The first half was meant to identify whether there was an effect of emotionality of assessment independent of the effects of other factors, the last half was meant to identify whether the words deemed emotional by participants were processed differently, and whether that mediating process explains some of the observed effects. Among each approach 3 analyses were done per each of the 3 components of interest.

Including valence normative assessments as a factor in the ANOVA analyses brings the problem of irregular distribution of valenced and non-valenced words between different levels of arousal, subjective significance and origin. The original set of words had the valence of stimuli aligned in groups, to make it as equal as possible, but as it was not manipulated in this study, the exact numbers of differently valenced words differed between groups (see S3 Appendix). This may become a troublesome issue, if we wanted to conduct analysis in a 3 x 3 x 3 x 2 model, in which the irregular distribution of valence would definitely play an important role in the shape of results. This is why we decided to conduct 3 analyses in a 3 (experimental factor level) x (valenced vs non-valenced) model, to reduce the influence of inequality between groups and control the valence factor in the analyses of other factors’ main effects.

In the P300 component we found the main effect of subjective significance (*F*(2, 66) = 2.97, *p* < .001, *η*_p_^2^ = .39), which is corresponding to the effect from the main analyses, however we also found the main effect of arousal to be significant in this component (*F*(2, 66) = 6.65, *p* = .002, *η*_p_^2^ = .17), with words low on arousal evoking higher amplitudes than the ones medium and high on arousal. We also found significant interactions between valence and arousal (*F*(2, 66) = 5.98, *p* = .004, *η*_p_^2^ = .15), as well as between valence and origin (*F*(2, 66) = 4.36, *p* = .02, *η*_p_^2^ = .12), however we did not find the interaction between valence and subjective significance, which further ensures the validity of effect evoked by subjective significance. In the P300 component we also found the main effect of valence (*F*(1, 33) = 9.47, *p* = .004, *η*_p_^2^ = .22), when arousal was controlled, with valenced words evoking higher amplitudes than the non-valenced ones, but we did not observe significant valence effects when other factors were controlled.

In the N450 component, when controlling the valence, we found main effects of origin (*F*(2, 66) = 16.94, p < .001, *η*_p_^2^ = .34) and subjective significance to be significant (*F*(2, 66) = 18.67, *p* < .001, *η*_p_^2^ = .36), both effects corresponding to the ones reported in the main analyses, however we also found main effect of arousal to be significant (*p* = .009), again with words low on arousal evoking more positive amplitudes—this effect was not found in the initial analyses. We also found significant interaction between emotional valence assessed in the normative study and subjective significance (*F*(2, 66) = 1.43, *p* < .001, *η*_p_^2^ = .24, as well as origin (*F*(2, 66) = 7.05, *p* = .002, *η*_p_^2^ = .18). Valence effect was significant when controlling the levels of arousal (*F*(1, 33) = 4.50, *p* = .04, *η*_p_^2^ = .12) and origin (*F*(2, 66) = 16.94, *p* < .001, *η*_p_^2^ = .34), with words assessed as valenced evoking more positive amplitudes.

In the LPC component we found main effects of all three experimental factors: origin (*F*(2, 66) = 8.12, *p* < .001, *η*_p_^2^ = .20), arousal (*F*(2, 66) = 6.21, *p* = .003, *η*_p_^2^ = .16) and subjective significance (*F*(2, 66) = 3.35, *p* = .04, *η*_p_^2^ = .09), however the pairwise comparisons did not reach statistical significance in the case of subjective significance. All the three effects have also been identified in the initial analyses. Furthermore, in the LPC component we found a significant interaction between valence and arousal (*F*(2, 66) = 4.35, *p* = .02, *η*_p_^2^ = .12), as well as main effects of valence when arousal was controlled (*F*(1, 33) = 4.83, *p* = .04, *η*_p_^2^ = .13), with valenced words evoking more positive amplitudes. All the exact results of analyses, with figures presenting significant differences, are presented in S3 Appendix.

The results of the analyses including normative assessments of words on the valence scale as factors have proven the validity of the significance effects observed throughout all the analyzed components, as well as the origin effects observed in the N450 and LPC components. However, these results have also outlined the strong relationship between valence and arousal of stimuli, which had been frequently mentioned in studies regarding emotions (e.g. [51]).

Finally, we would like to report the analysis of participants’ decisions concerning emotionality. This would help us to establish whether decisions explain the observed differences in amplitudes, or whether the observed effects should be interpreted solely as stemming from the explicit processing of the word’s meaning and emotional connotations. Here, we could encounter the same problems as with analyses including normative valence ratings: increasing the level of arousal should lead to more emotional decisions, thus making it hard to interpret the main effect of emotional decision. We opted to again control one other factor at a time when comparing trials with detected emotional vs neutral words stimuli.

At the P300 component in all three analyses, there was an effect of emotional decision (*F*(1, 33) = 3.11, *p* < .001, *η*_p_^2^ = .48), such that words deemed emotional produced more positive amplitudes than non-emotional words. This shows the early attentional capture of words relevant to the task of emotional categorization. Nonetheless, we replicated the effect of subjective significance when controlling for emotional decisions in this component (*F*(2, 66) = 3.83, *p* = 0.03, *η*_p_^2^ = .10). However, we also observed a main effect of arousal with the emotional decisions controlled (*F*(2, 66) = 4.65, *p* = .01, *η*_p_^2^ = .12), with highly arousing words evoking higher amplitudes, as well as an effect of interaction between emotional decision and origin (*F*(2, 66) = 3.38, *p* = .04, *η*_p_^2^ = .44).

At the N450 component in all three analyses, there was the main effect of emotional decision (*F*(1, 33) = 23.07, *p* < .001, *η*_p_^2^ = .41), with words deemed emotional producing more positive amplitudes than those deemed neutral. We replicated the effect of subjective significance (*F*(2, 66) = 4.61, *p* = 0.01, *η*_p_^2^ = .12), but we did not observe an effect of origin when controlling for whether the words were deemed emotional.

Lastly, at the LPC, we observed a main effect of emotional decision in all three analyses (*F*(1, 33) = 1.07, *p* = .003, *η*_p_^2^ = .23), words deemed emotional produced more positive amplitudes than those deemed neutral. What is intriguing is that we did not see the effects of subjective significance or origin, when controlling for emotional decisions, that we saw in the main analysis. However, again, we observed the effect of arousal (*F*(2, 66) = 3.67, *p* = .03, *η*_p_^2^ = .10), with words low on arousal evoking higher amplitudes than the ones medium on this factor. All the exact results of analyses, with figures presenting significant differences, are presented in S3 Appendix.

**Fig 10:**
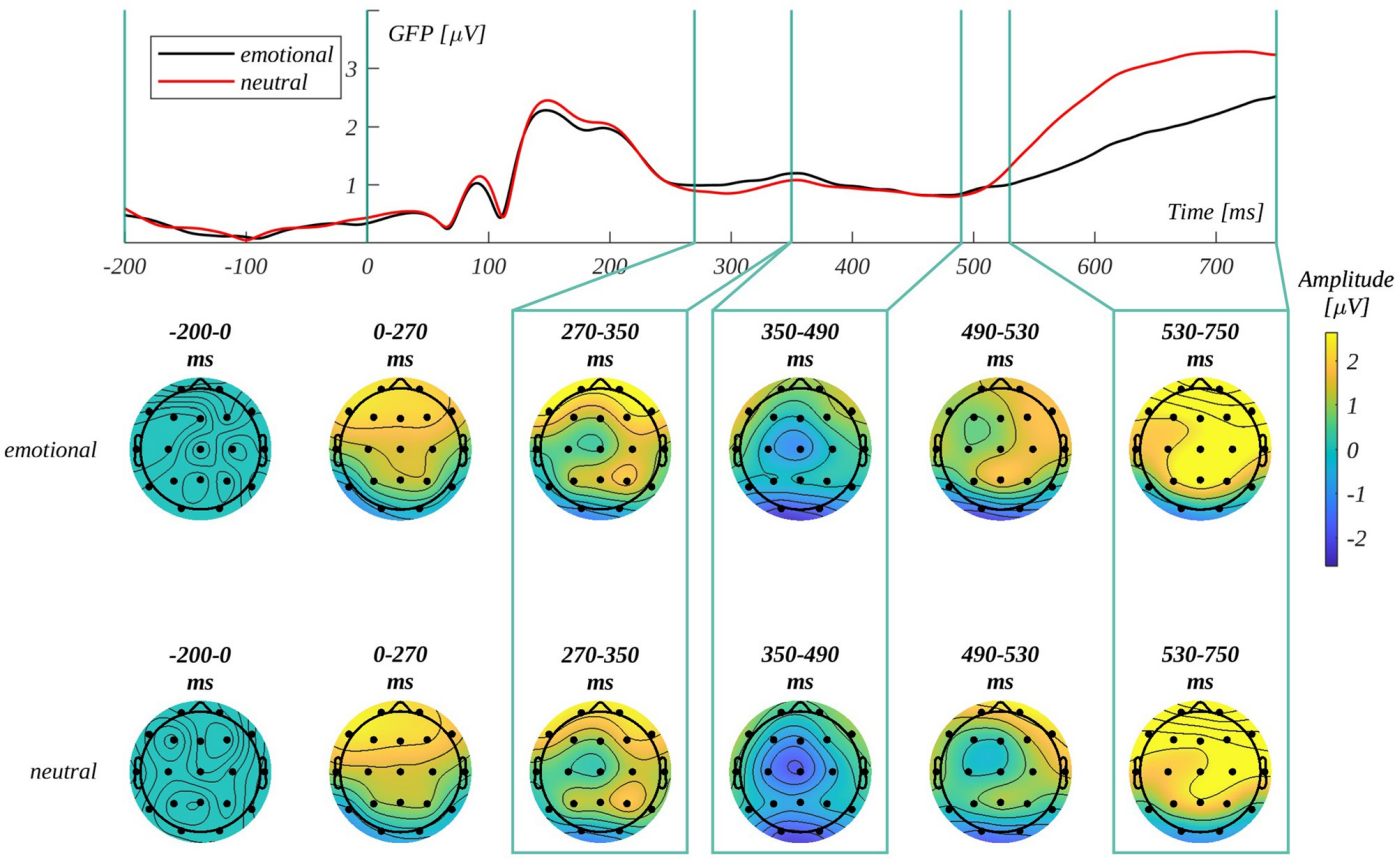
Analysis of global field power. Top—Global Field Power (GFP); middle and bottom—topographical distribution of mean potential in consecutive time windows for emotional and neutral stimuli as assessed by the participants in the case of emotional and neutral stimuli respectively.

When we use the decisions themselves as a factor in the analyses, we can see different stages of the decision-making process in different components (Fig 10): P300, the early stage, when the decision is processed [56–58], after which processing is mostly influenced by the decision itself, which can be seen in analyses of N450 and LPC components. The results in the P300 and N450 components confirm the findings from the initial analyses regarding subjective significance, indicating its’ influence on both decision making and word processing (to which is the N450 component related; [48, 59]). The effect of arousal in the LPC component could be interpreted as a marker of assessing the relevance of stimuli to the task [60], however it also once again indicates the strong relationship between arousal and the concept of emotionality itself [51, 61]. The lack of replication of effects of origin (N450 and LPC) may stem from the difference in how much more frequently words of automatic origin are deemed emotional than those of reflective origin. This in itself is an interesting result, showing how much the automatic vs. reflective processing of words is intimately related with judgements of emotionality.

## 4. Discussion

### 4.1. Behavioural results

In the current experiment, we sought the role of activation factors and origin of affect in the emotional categorisations of words. The behavioural results for reaction times confirmed the hypothesis of subjective significance being an indicator of the activation of reflective processing [7, 11, 12]. Not only did high subjective significance itself lengthen the reaction time, when analysing the interaction with origin we found that the effect appeared particularly within the group of words of reflective origin. This effect should be paired with the lack of influence of arousal on reaction times. The arousal effect could have been expected in this experiment, considering previous studies [2, 3]; however, with the orthogonal manipulation of subjective significance, this effect turned out to be non-existent. The procedure employed in this experiment may be partially responsible for this shape of the effects—as the participants had to assess the emotionality consciously, the consideration was strongly involved in assessments; thus, the effect was present for reflective stimuli. We did not observe the effects of origin related to reaction times, which may be perceived as standing in contradiction to previous results [16–19] obtained for different paradigms. However, in the current experiment, we did not expect the effects of origin, mainly because of the strong influence of both arousal and subjective significance as activation factors for both automatic and reflective evaluative systems.

The fact that emotional arousal did not influence reaction times in this task could also be interpreted in the context of particular stimuli picked for this experiment. Valence and arousal are factors of emotional processing which are strongly correlated [4, 5, 51, 61], and as we tried to align the valence among experimental groups, it was nearly impossible to use extremely arousing or non-arousing words. In fact, we were not able to include any extremely arousing words in the experiment (value above 6.5 from the normative study, [51]). This may be responsible for not observing an arousal effect in reaction times, there may have been not enough arousing words to lengthen the reaction times in the groups labelled with high arousal.

The emotionality ratings for words increased in general with increasing levels of arousal and subjective significance, which supports the idea of these two dimensions being activation factors for automatic and reflective processing, respectively [12, 48, 51]. The automatically originated words were assessed as more emotional than the reflective ones, which is a bias that probably comes from the perception of the concept of emotionality in the general population—emotions regarding deprivation of basic needs and basic pleasurable states (automatic originated) are perceived as being more in line with the stereotype of emotion than those regarding more complex social concepts (reflective originated) [9, 10]. It is also worth noting that, within the group of automatically originated words, the emotionality ratings did not differ depending on the arousal level, but rather they were differentiated based on significance level. This interaction between the factors shows the bias of perceiving automatically originated words as more arousing in general and thus not differing on the arousal scale.

### 4.2. EEG results

At the electrophysiological level, we analysed three components of interest: P300, N450 and LPC. For the P300 component, we observed a main effect of subjective significance. The amplitude became significantly more positive with an increase in levels of subjective significance. This seems to be a very intuitive result, as the subjective assessment of the stimuli’s relevance would change with the level of significance; as already mentioned, P300 was hypothesised to be closely linked to the processing not only of stimuli in the perspective of updated context (what is relevant and what is not) but also in the meaning of their future consequences [34]. Furthermore, we obtained an interaction between origin and subjective significance, such that the P300 mean amplitude increased alongside the level of subjective significance only for stimuli of automatic and null origins. While the main effect shows that subjective significance is rapidly detected after word onset and modulates ERP components earlier than other factors, the interaction indicates that this detection is slower for reflective stimuli, as reflective words did not exhibit the modulation of P300 associated with subjective significance. It is worth mentioning that we did not observe a main effect for arousal, which is also a highly relevant factor in this task and has been considered faster and more automatic than subjective significance. However, not identifying the arousal effect may also be interpreted by the lack of extremely arousing words among the experimental stimuli, similarly to the absence of arousal influence on reaction times. Nevertheless, the subjective significance seemed to be processed faster (already at the P300 time range) and additionally modulated by its interaction with origin. The effect of subjective significance in the P300 component was confirmed when valence of emotional words was included in the analyses, as well as when the decisions made by participants were included as a factor, which further underlines the importance of subjective significance on early stages of decision-making process. What is more, origin of emotion came into interaction with both valence and decisions, again showing the modulating role of origin in this component.

As for the N450 component, we also found a main effect for subjective significance—the amplitude became progressively less negative with the increase in the levels of subjective significance. This result confirms our hypotheses and is in line with previous findings [12, 48, 50] that indicated people can be susceptible to different levels of subjective significance in word stimuli. Like P300, the role of N450 potential is associated with the processes of the evaluation of stimuli (in relevance to one’s self) to distinguish the significant from the non-significant ones. While analysing the differences in the N450 component, we also obtained a main effect for origin, in which the N450 mean amplitude became more negative alongside changes in the level of origin (starting from automatic, through null to reflective). It therefore seems that deciding between emotional and non-emotional categories for words produced the most conflict for groups of reflective words compared to those of automatic and null origin; this effect was probably because the decision required more cognitive effort in processing for reflective words and was not as intuitive as for automatic ones. Both the effects of subjective significance and origin were confirmed in analyses with the values of valence controlled (the analyses also yielded interactions between these factors and valence), however the analyses including decisions as factor confirmed only the effect of subjective significance. This may suggest, similarly to the interactions observed in the previous component, that the origin modulates the processing of emotional properties of stimuli on early stages of decision-making process, before the decision itself was made.

Summarising the effects of these components, we note that the effect of subjective significance started the earliest, as early as the P300 component, and this modulation continued throughout the N450 component with the same shape, which may suggest that the modulation based on subjective significance was produced by a longer, more global process that facilitated a fast recognition of word significance. The main effect of origin started later, at the N450 component, where words of reflective origin were processed in a way that indicated more conflict. The special way that reflective stimuli are processed could, however, be seen earlier, as the effect of subjective significance on P300 was strangely absent for this level of origin. Although arousal had a similar effect on emotionality ratings as the other two factors, we did not observe any modulation associated with this factor for components occurring before the LPC. Three interpretations may shed light on this result. First, the absence of extremely arousing stimuli, which was already mentioned in the context of behavioral results. Secondly, when manipulating all three factors, arousal may not have stood out as a factor capturing early attention in emotionality judgements, thus changing the judgements only through an affect induced later in the evaluation. A third hypothesis is that the processing of stimuli arousal, more so than other factors, may have been conducted by the basal brain structures, such as the amygdala [62], whose activity cannot be directly measured by scalp EEG, and whose projection to the cortex was not sufficient to affect the EEG potential significantly.

The last component (LPC) is the one most associated with explicit processing of emotionality [22, 40], and thus, as predicted by the explicit judgements of emotionality, we observed emotional modulation by all three factors. First, the main effect of origin mirrored the pattern seen throughout the emotionality ratings, of larger amplitude for more automatic words. The main effects of the activating factors—arousal and subjective significance—both exhibited a more positive amplitude for the high-level condition compared to the medium-level condition. These results demonstrate that these factors produce substantial emotional modulation independent of themselves and, more importantly, independent of the level of valence, which was confirmed in the analyses including valence as a factor in analyses In this regard, the present experiment corroborates the results of previous studies [19, 42], while being the first to compare how an orthogonal manipulation of all three factors affects ERPs in emotionality judgements. This provides encouraging evidence for the need for and direction that an expansion of our understanding of the emotional load of stimuli and emotionality judgements might take [43].

Examining the results in greater detail, we note that the pattern of differences in amplitude for arousal and subjective significance did not exactly match their emotionality ratings, as indexed by their lack of differentiation at low levels. An inspection of their associated interaction effects may provide an explanation, particularly the interaction between subjective significance and origin. For automatic origin, words of both low and medium level of subjective significance produced a less positive amplitude than those with a high level of this factor. However, for reflective origin, words with both a low and high level of subjective significance produced a more positive amplitude than words of medium significance. Averaging these effects explains the lack of differentiation at low levels of subjective significance, while a more thorough view stratifying by different origins reveals a difference in how subjective significance is processed for words of automatic and reflective origin, which could not be seen with behavioural measures alone. Stratifying this effect by the levels of subjective significance also reveals that this change in pattern is driven by the fact that words with a low level of subjective significance are not modulated by origin. These effects may be jointly explained by the existence of an attentional capture produced by reflective words with low subjective significance, which can be seen in the LPC, but that does not lead to an increase in the judgements of emotionality. As these two factors have not yet been studied together, we cannot compare these results with prior studies. However, we can compare this with previous components, and note that this effect continues a pattern of such interactions seen earlier, as when reflective origin delayed the onset of the significance effect.

The exploratory analyses have revealed two main phenomena, that we were not able to identify in the general analyses. First, it emphasized the interaction between valence and arousal, that could have been responsible for the absence of valence effects in the general analyses, which was discussed earlier. Secondly, the type of decision that was made was a factor influencing post-decision processing in a stronger way than the emotional factors, which could be seen in the effects regarding N450 and LPC components. The effects of subjective significance and origin observed in the main analyses turned out to be insignificant when the decisions were included as a factor. This inequality in observed effects underlines the difference between the role of early and late components in the decision-making process [56–58, 60].

### 4.3. Limitations

The simplicity of the procedure employed in this experiment may be perceived as a limitation of this study. As the procedure motivated participants to engage in conscious processing of word meaning, we could have missed a chance to capture the effects regarding implicit processing, especially in the EEG measures. On the other hand, similar procedures have frequently been employed in experiments [63, 64], as the simplicity makes it possible to sort out any potential disrupting factors interacting with the independent variables. The study also did not explore the effects for factors differing with valence, as all of the factors were aligned to moderate levels of this factor. However, we explored the exact relationship between arousal, subjective significance and different levels of valence in our previous experiments [19, 50]. It should be mentioned, that valence of words was aligned between different groups of stimuli, however the proportion of valenced (positive or negative) and non-valenced (neutral) words was not equal between groups (see S1 Appendix). This is a limitation, deriving from manipulating three other experimental factors simultaneously. Aligning valence between stimuli groups also resulted in a small number of low-arousing words and the lack of extremely arousing words among the experimental stimuli, which is also a limitation, as it could have affected the results of the study. Finally, the experiment was conducted on young, healthy adults, which makes it difficult to extrapolate the results onto the entire population. However, this selection of the experimental group made it possible to gather precise and internally consistent EEG recordings and construct a valid manipulation of the subjective significance variable.

Another limitation of the present study comes from the entangled nature of dimensional conceptions of emotional load of stimuli. As many factors studied in the literature, such as valence, arousal, and concreteness all exhibit complex, and sometimes non-linear dependencies [51], it is hard to control all relevant factors when scaling up the experiments to long lists of words, as was done in the present study. This stems from the fact that for some combinations of arousal, origin and subjective significance levels, the distribution of words may be so sparse in, for example, medium concreteness, that there may not be enough words to construct a list of experimental stimuli with enough stimuli in each group, controlling for concreteness. This is the reason that we were not able to control for concreteness of the words in the present study, a fact which has sometimes been shown to modulate how the word is processed, and consequently what ERPs it produces [20, 46]. Another important limitation is the potential confounding role of valence. It has been widely reported in studies measuring affective qualities of words, that an increase in arousal ratings is associated with an increase in the variance of valence ratings, producing a curvilinear relationship [51]. This presents a challenge for the selection of stimuli. While we were able to match each of the 27 experimental conditions on mean valence ratings, the conditions varied in valence SDs (as reported in S1 Appendix). We took care to analyse whether this relationship had a confounding effect on the observed EEG effects, the results of which suggested it did not. We cannot however rule out that valence influenced behavioural results, a matter which warrants further research.

Lastly, it may be argued that our strategy to pick mostly stimuli neutral on valence may have influenced the participants in the execution of the task of emotionality judgements, valence of the word being the primary component of word emotionality. About 28% of our words had high or low valence (as operationalized in the results section), which may have influenced the results by making the "emotional" response more rare. One way to overcome this limitation would have been to pick the same number of valence and non-valenced words. However, the advantage of our stimuli is that they exhibit a more real-world-like distribution of emotionality between words, because words with such valence ratings constitute 27% of nouns in the affective norms database we used.

### 4.4. Conclusions

In the current experiment, we showed the validity of an approach based on orthogonal manipulations for emotional factors in word stimuli. It appeared that subjective significance shaped behavioural results and all stages of word processing indexed in EEG measures. Importantly, we showed that subjective significance and reflective origin were associated factors as indexed in behavioural results, but also having their specificity resulting in opposite patterns of results, as indexed in the N450 component. This supports the validity of the approach differentiating between activation (intensity of emotional stimuli) and the content of the stimuli (i.e. type of processes leading to an emotional reaction). Exploratory analyses have indicated the strength of relation between valence and arousal, as well as the differences in processing emotional factors in early and late stages of decision-making. Our experiment showed that emotionality is not only related to positive or negative valence (cf. [1]), and we demonstrated that activating factors for words aligned in valence result in similar effects as for extremely valenced stimuli [65].

## Supporting information

S1 Appendix (XLSX)

S2 Appendix (XLSX)

S3 Appendix (XLSX)
